# Supplementary material for: A Bidirectional Non-Coding RNA Promoter Mediates Long-Range Gene Expression Regulation
Source: Genes (Basel). 2024 Apr 25;15(5):549. doi: 10.3390/genes15050549 (PMC11120797; doi:10.3390/genes15050549)
Supplement: Supplementary file 1 [file genes-15-00549-s001.zip › Supplementary_Material_final/Supplementary_File_S7.pdf]

genes

MDPI

Article

A bidirectional non-coding RNA promoter mediates long-range gene expression regulation

Carlos Alberto Peralta-Alvarez[1,2], Hober Nelson Nuñez-Martinez[1], Ángel Josué Cerecedo-Castillo[1], Augusto César Poot-Hernández[2], Gustavo Tapia-Urzua[1], Sylvia Patricia Garza-Manero[1], Georgina Guerrero[1], Félix Recillas-Targa[1]

Supplementary File 7: Supplementary Figures.

Figure S1

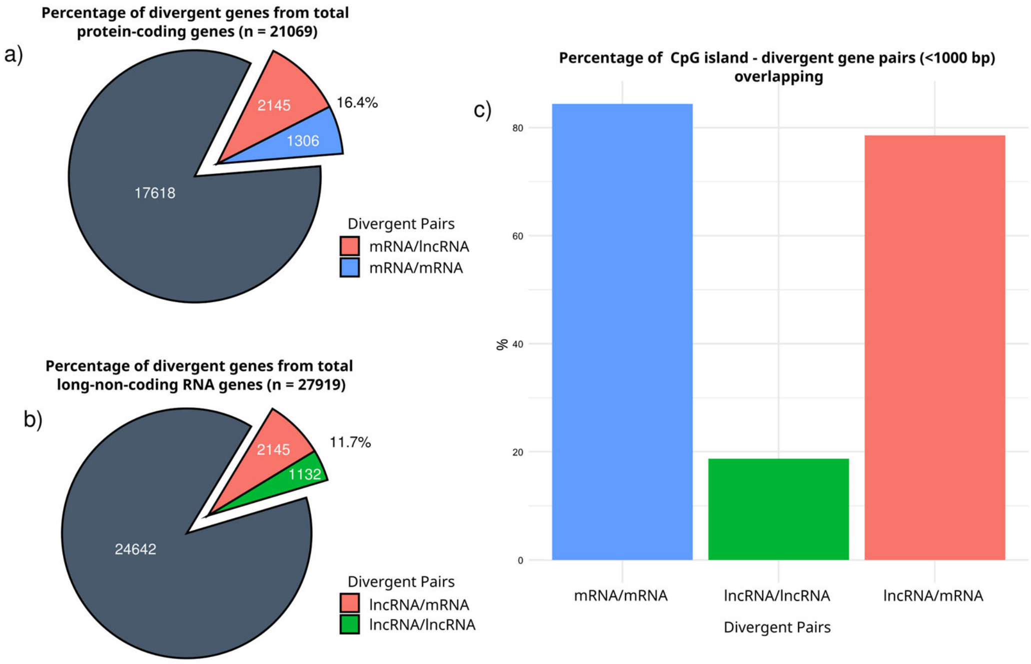

Figure 1S. Divergent genes representation on human genome. **a**) Percentage of divergent protein-coding genes separated by 1000 bp or less relative to total Hon 2017 protein-coding human genes. b) Percentage of divergent long-non-coding RNA genes separated by 1000 bp or less relative to total Hon 2017 long-non-coding RNA human genes. **c**) Intersection percentage of divergent genes up to 1000 bp with UCSC table browser hg19 CpG island data set.

Figure S2

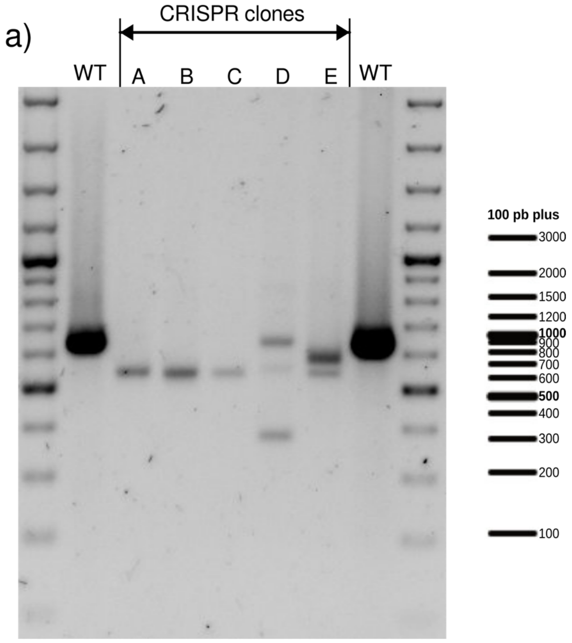

Figure 2S. CRISPR-Cas9 clones from BBQ elimination assay. **a**) PCR genotypification for CRISPR-Cas9 assay targeting BBQ element, lanes marked as A,B and C are apparent biallelic mutant clones, lane D appears to be a monoallelic BBQ elimination along a probable insertion event, and lane E appears to be a heterogeneous biallelic mutant with small deletions compared to the wild-type control. Mutant clones B1,B2, T1 or T2 are not present on this gel.

Figure S3

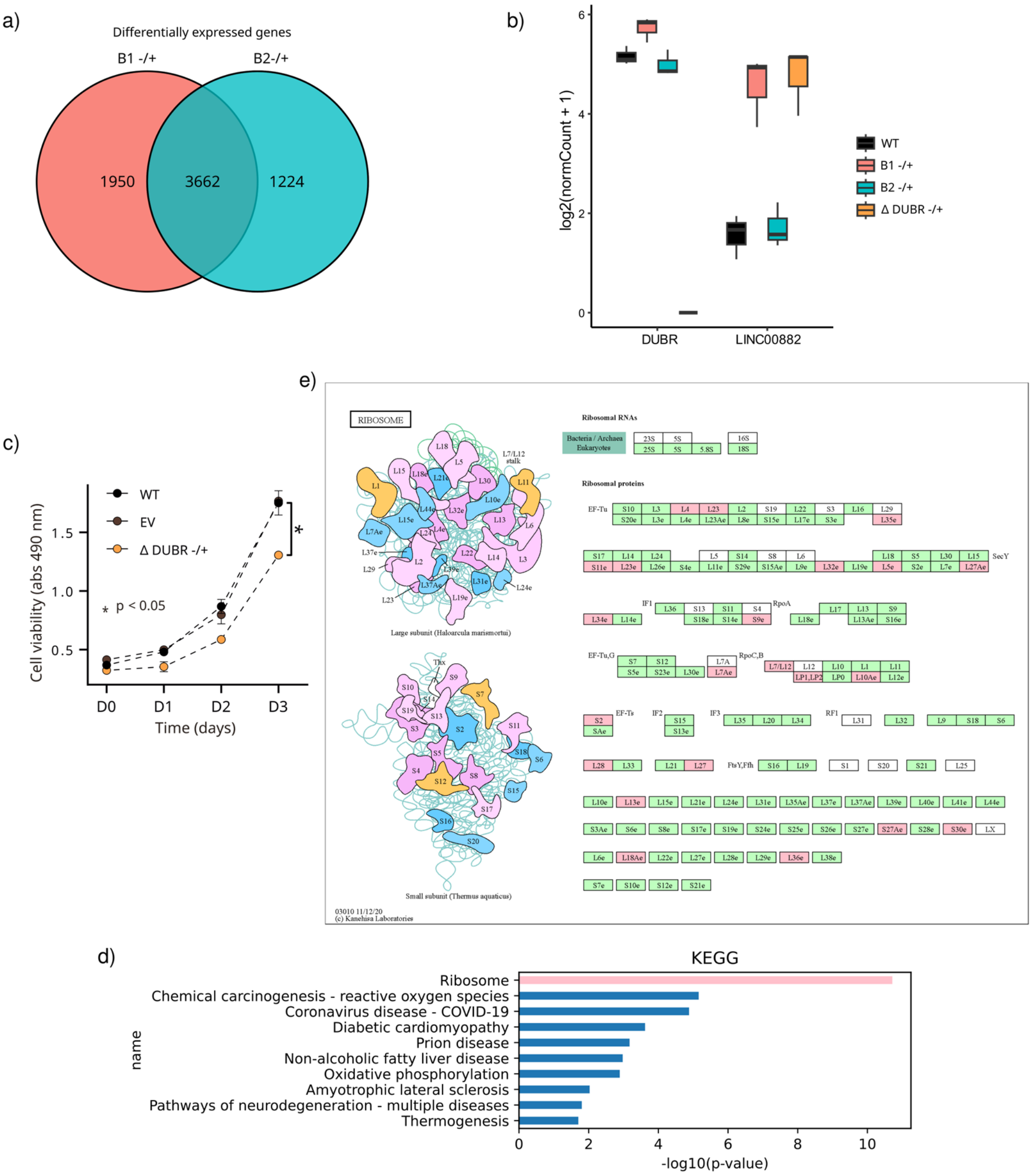

Figure 3S. Differentially expressed genes and affected cell pathways upon BBQ elimination. **a**) Venn diagram to show the intersection of differential expressed genes of B1 and B2 clones independently compared to wild-type control. **b**) DESeq2 normalized counts for DUBR and LINC0082 on K562 wild-type, BBQ mutant clones and DUBR mutant clone. **c**) MTT proliferation assay for BBQ mutant clone, significance is measured by T-test (p-value < 0.05 = *). **d**) KEGG pathways enriched by GO terms on the list of genes that are differentially expressed on DUBR mutant and BBQ mutant clones. **e**) KEGG human ribosome pathway map (hsa03010); human genes are highlighted with green boxes and differentially expressed genes upon BBQ elimination on both DUBR and BBQ elimination assays are marked with red boxes.

Figure S4

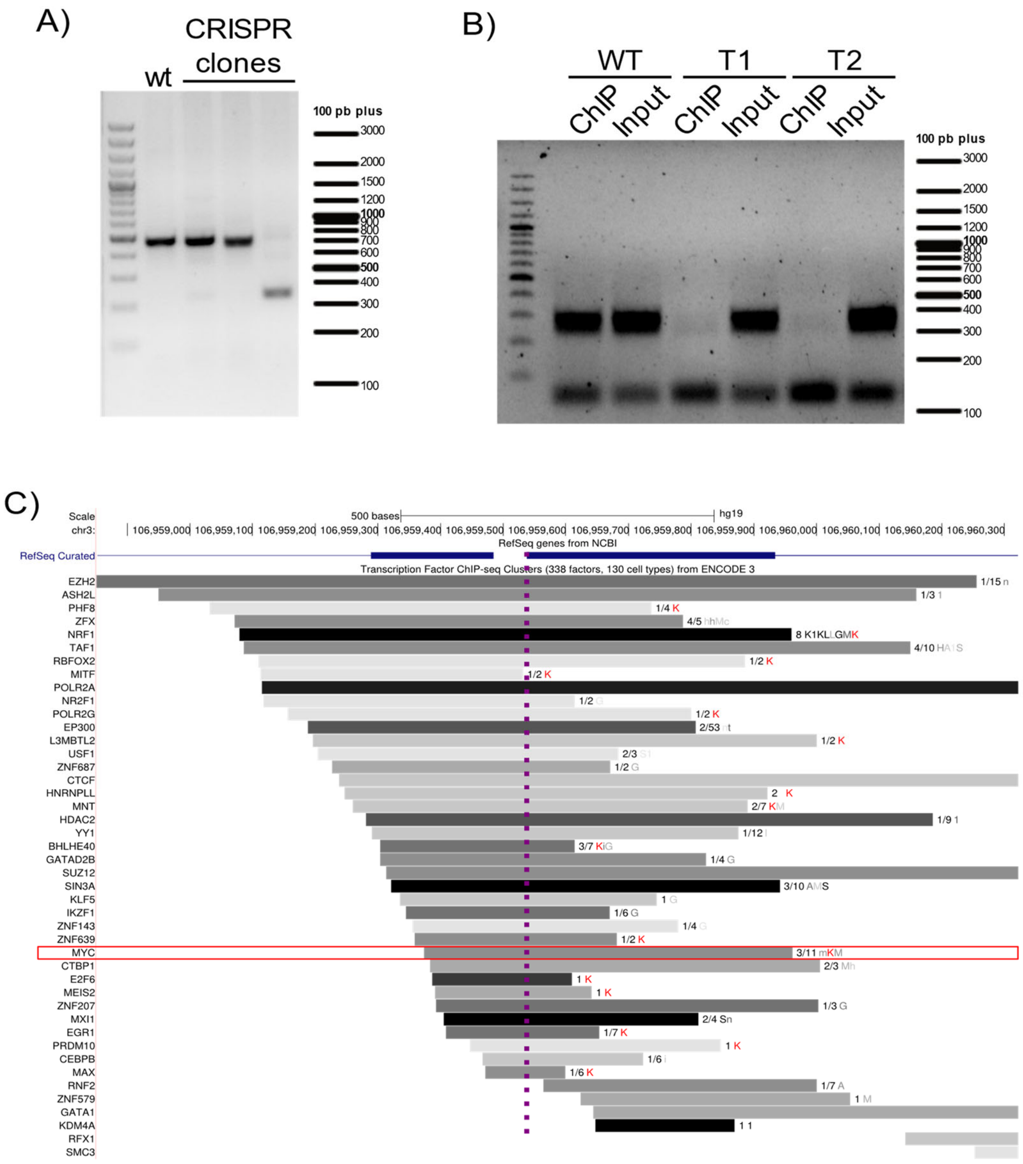

Figure 4S. CRISPR-Cas9 elimination of DUBR canonical TSS region. **a)** PCR genotypification for CRISPR-Cas9 assay targeting DUBR TSS region. b) ChIP PCR for c-Myc on DUBR TSS mutant clones. c) ENCODE 3 Transcription binding sites measured by ChIP-Seq, red “K” indicates a peak or more in K562 cell line, dashed purple line overlaps *DUBR* TSS, (retrieved from UCSC Genome Browser website).

Figure S5

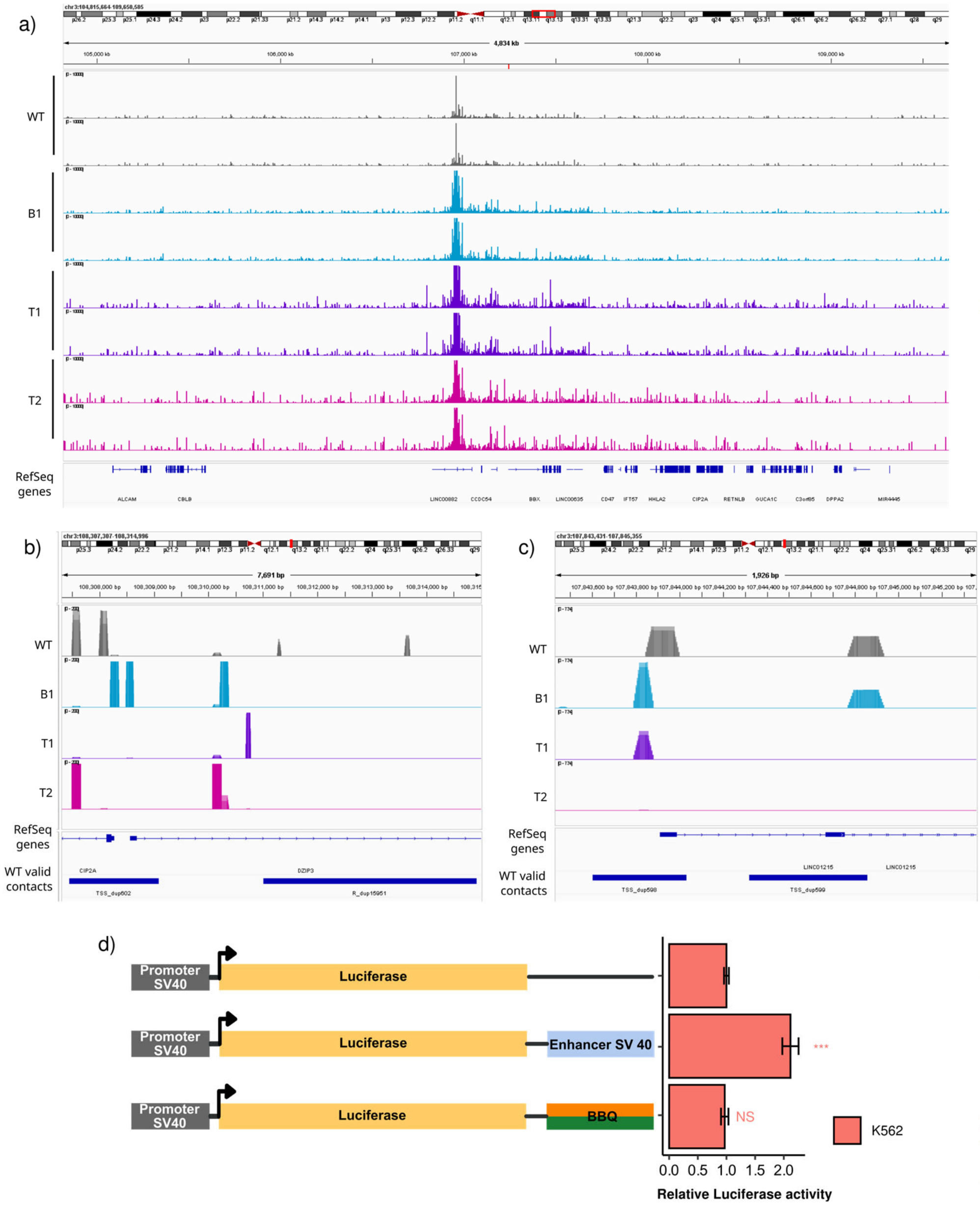

Figure 5S. BBQ 4C-Seq interaction changes over distal gene TSS upon BBQ mutations. **a**) Scaled and normalized 4C-Seq signal of BBQ viewpoint assay for B1, T1, T2, and WT cells over a region of 4.8 mb around BBQ element. The signal is shown at a linear scale, and all tracks use the same data range interval. **b** and **c**) Scaled and normalized 4C-Seq signal for B1, T1, T2, and WT cells over genome segments near CIP2A and DZIP3, (d) and RPL13P8 (E) TSS (+/- 3 kb from TSS). The signal is shown at a linear scale, and all tracks use the same data range interval. d) Luciferase activity of cloned sequences expressed relative to pGL3-promoter (SV 40) vector on K562 cell line (n = 6). Significant differences of each construct versus pGL3-promoter vector (pGL3 with SV40 promoter) was tested using Mann-Whitney-Wilcoxon test (p-value < 0.0001 = ***, p-value < 0.001 = ** and p-value < 0.01 = *).

Figure 6S

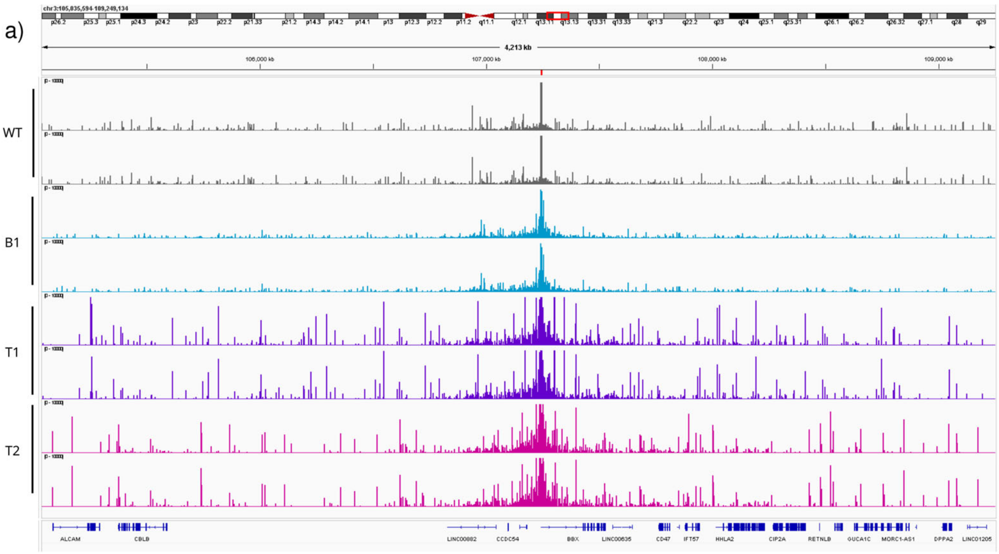

Figure 6S. BBX 4C-Seq general overview. **a**) Scaled and normalized 4C-Seq signal of BBX viewpoint assay for B1, T1, T2, and WT cells over a region of 4.2 mb around BBX element. The signal is shown at a linear scale, and all tracks use the same data range interval.
